# Regulation of endogenous dsRNA: strategies for self-tolerance

**DOI:** 10.1098/rsob.260111

**Published:** 2026-11-04

**Authors:** Jacki Heraud-Farlow, Yu-Pei Chan, Sophia Davidson, Carl Walkley

**Affiliations:** ^1^ Centre for Innate Immunity and Infectious Disease, Hudson Institute of Medical Research, Clayton, Victoria, Australia; ^2^ Monash University—Clayton Campus, Melbourne, Victoria, Australia; ^3^ Molecular Sciences Division, Walter and Eliza Hall Institute of Medical Research, Melbourne, Victoria, Australia

**Keywords:** innate immunity, RNA modification, RNA, type I interferon, double-stranded RNA, autoinflammation, self-tolerance

## Abstract

Cells must continuously distinguish cellular-derived RNA from pathogenic RNA to mount effective antiviral responses while avoiding harmful autoinflammation. Cytoplasmic RNA-sensing receptors, including RIG-I-like receptors, PKR, OAS-RNase L and ZBP1, detect RNA primarily through molecular features such as structure (double- versus single-stranded), modification state and subcellular localization rather than sequence. While these are characteristics of viral nucleic acids, mammalian genomes generate abundant self-derived double-stranded RNA (dsRNA), including from transposable elements, antisense transcription and RNA processing intermediates, which exist in the cytoplasm. This review describes our current understanding of how cells achieve self-tolerance through the active regulation of endogenous RNA. These mechanisms comprise epigenetic silencing of repetitive elements, RNA modifications (such as A-to-I editing and m^6^A), precise splicing, regulated nuclear export and decay, circRNA buffering, protein shielding and dedicated RNA degradation pathways. Insights from rare inherited monogenic interferonopathies and murine models illustrate how perturbations in these pathways lead to inappropriate activation of cytoplasmic RNA sensors and inflammatory disease when cellular dsRNA is mistakenly recognized as non-self. We highlight how dysregulated dsRNA homeostasis contributes to common autoimmune and neuroinflammatory disorders. Understanding how cells regulate endogenous dsRNA is crucial for defining disease mechanisms and developing new therapeutic strategies.

## 1. The RNA problem

Genomic DNA contains the information plan for an organism and is strictly compartmentalized within the nucleus and mitochondria. Consequently, the presence of cytosolic DNA is fundamentally aberrant, signalling cell damage, cell death or a DNA pathogen and demands an immediate response regardless of origin [1]. Gene expression requires transcribing DNA into ribonucleic acid (RNA) species that travel from the nucleus to the cytoplasm. Beyond canonical messenger RNAs (mRNAs), transfer RNAs (tRNAs) and ribosomal RNAs (rRNAs), a vast proportion of the genome is transcribed into non-coding species, including long non-coding RNAs (lncRNAs), microRNAs and circular RNAs (circRNAs). The obligatory export of host RNA creates a core immunological challenge: distinguishing ‘self’-derived cytosolic RNA from foreign viral nucleic acids [2]. This becomes more complex in primates where retrotransposons, such as *Alu* elements that comprise up to 11% of the human genome, can form long double-stranded RNA (dsRNA) structures transcribed as inverted repeats. The evolutionarily conserved cytoplasmic nucleic acid sensing system (also known as the innate immune sensing system) uses molecular features of nucleic acids, rather than specific sequence motifs, to enable ‘self’ versus ‘non-self’ discrimination [2–5]. This system needs to discern the cell's own RNA from potentially damaging RNA pathogens.

## 2. Sensors of RNA

Cells express a diverse set of pattern-recognition receptors (PRRs) that survey RNA based on its structure (e.g. single- versus double-stranded), length, chemical modification and subcellular localization (figure 1) [2]. RNA surveillance occurs predominantly within cytosolic and endosomal compartments, with additional layers of regulation imposed by nuclear RNA processing and quality control mechanisms. While individual RNA-sensing PRRs are specialized to detect distinct classes of RNA, there is a degree of redundancy in the ligand-binding preferences. Importantly, immunogenic RNAs are not solely of pathogen origin, but can also arise from host genomic material [6–9], particularly under conditions of cellular stress. While RIG-I–like receptors (RLRs) and toll-like receptors (TLRs) act as primary drivers of type I interferon (IFN) and pro-inflammatory cytokine expression, other sensors trigger complementary stress responses, including translational arrest (PKR), targeted RNA cleavage (OAS-RNase L) and programmed cell death (ZBP1) (figure 1 and table 1).

**Figure 1.**
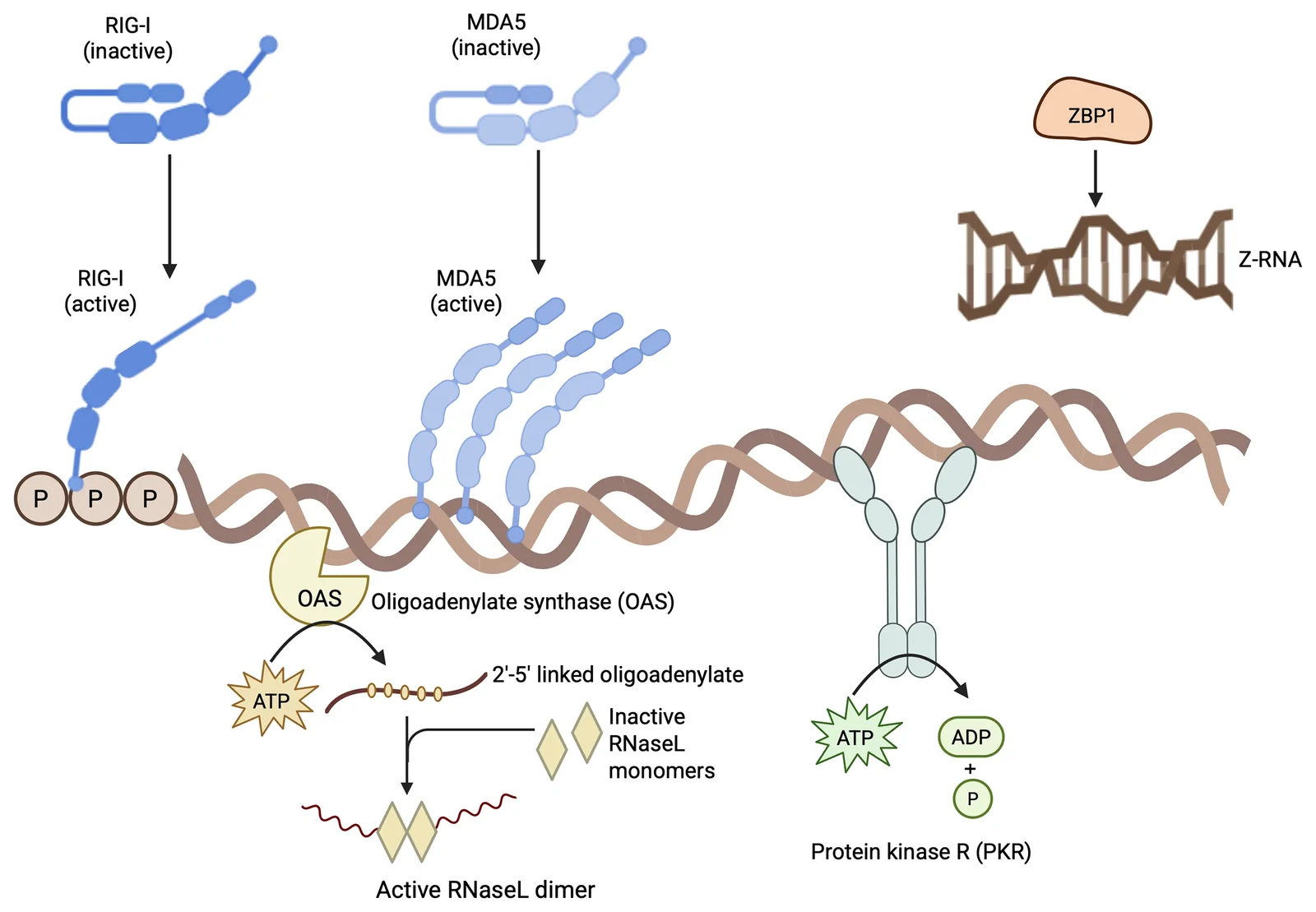
Cytoplasmic RNA sensors survey different aspects of RNA. The different cytoplasmic RNA sensors assess/detect/interact with/bind to different characteristics of RNA to determine their response. RIG-I preferentially detects short dsRNA molecules bearing a 5′ triphosphate (5′ppp) or diphosphate group (5′pp). MDA5 binds and assembles into filaments that are particularly stable on long regions of dsRNA duplexes. PKR has a biochemical preference for shorter dsRNA substrates and, upon binding, undergoes dimerization and *trans* autophosphorylation to activate its kinase activity. OAS-RNaseL is distinct from the other sensors illustrated in that it is a two-part signalling mechanism. OAS1, 2 and 3 proteins have a varied substrate preference, which may be reflective of the number of OAS domains each has: OAS1 interacts with shorter dsRNA (approx. 18 bp); OAS2 requires a minimum of approximately 35 bp dsRNA for activation, while OAS3 binds to approximately 40–60 bp lengths of dsRNA. Upon binding, OAS proteins oligomerizing ATP into 2′–5′-oligoadenylates (2–5A), a second messenger that activates RNaseL. ZBP1 binds to Z-form dsRNA, which is an atypical high-energy, left-handed double-helical conformation of dsRNA that is distinct from most RNA in the cell.

**Table 1.** Summary of dsRNA sensors and their properties.

**dsRNA sensor protein**	**substrates/ligands (where known)**	**mouse models**	**associated diseases / functional impact**
**RIG-I** *(encoded by DDX58)*	— preferentially binds dsRNA bearing a 5′ppp or 5′pp group, with blunt-ended molecules being the most immunogenic [10–15] — excludes host mRNAs via steric exclusion if they possess a 2′-O methylation at the 5′-terminal nucleotide — mutant RIG-I shifts substrate preference towards host small non-coding Y-RNAs (prominently RNY4) [16]	— *Ddx58*-deficient (RIG-I knock-out) mice: one reported allele exhibits high embryonic lethality [17], while a second allele yields viable deficient mice [18] — point mutant mouse model expressing a disease-associated variant of RIG-I [16]	— activates MAVS [19], leading to downstream activation of TBK1, IKKε, IRF3, IRF7 and NF-κB to drive type I IFN and inflammatory gene expression — germline variants are identified in patients with autoimmune/autoinflammatory conditions, including Singleton-Merten syndrome, Aicardi-Goutières syndrome, systemic lupus erythematosus (SLE) and paediatric neuroinflammatory syndromes [20–22] — the mutant RIG-I mouse model develops a lethal nephritis phenotype in the kidney [16]
**MDA5** *(encoded by IFIH1)*	— preferentially senses long dsRNA duplexes [10,15] — binds sequence-independently via the phosphodiester backbone to form cooperative filaments — cellular/endogenous ligands include structured RNAs derived from inverted *Alu* elements [23] — binds high-molecular-weight viral RNA fractions containing both single- and double-stranded segments (e.g. from picornaviruses like EMCV) [24,25]	— *Ifih1*-deficient (Mda5 knock-out) mice: viable and largely normal when housed conventionally and unchallenged [10] — gain-of-function polymorphism/point mutant mouse models that mimic patient genetics by expressing variant proteins and develop inflammatory phenotypes in mice, including autoimmune myocarditis and nephritis [26–29]	— drives type I IFN and inflammatory responses via cooperative filament assembly and MAVS signalling; uses the same downstream signalling as RIG-I — germline variants are reported in patients with Aicardi-Goutières syndrome, Singleton-Merten syndrome, SLE and paediatric neuroinflammatory syndromes [8,22,30,31] — genetic variants demonstrate a fitness trade-off: loss-of-function variants protect against type I diabetes, psoriasis and hypothyroidism, but increase the risk for inflammatory bowel disease owing to reduced viral defence [29,32,33]
**LGP2** *(encoded by DHX58)*	— binds dsRNA but lacks independent signal-transducing capacity because it lacks N-terminal caspase activation and recruitment domains (CARDs) [34,35]	— primarily evaluated as a regulatory host cofactor within viral and cellular dsRNA-sensing contexts	— acts as a structure- and localization-sensitive cofactor that directly interacts with and modulates RIG-I and MDA5 activation to tune responses according to dsRNA topology and cellular context [34,36]
**PKR** *(protein kinase R, encoded by EIF2AK2)*	— senses short dsRNA duplexes of >33 base pairs (bp); can engage dsRNAs shorter than 33 bp if flanked by single-stranded regions of 10–15 nucleotides. With maximal activation observed for duplexes ≥79 bp — sequence-independent recognition that requires contact with 2′-hydroxyl groups within the minor groove of A-form RNA; tolerates mismatches and bulges — activation is enhanced by a 5′-triphosphate moiety on RNAs with limited secondary structures [37] — triggered by viral genomes across ssRNA, dsRNA and dsDNA viruses — endogenous substrates include non-coding retrotransposons (such as *Alu* elements), mitochondrial RNAs, small nucleolar RNAs and transfer RNAs [38,39] — bound and suppressed under homeostatic conditions by short, imperfect endogenous circular RNAs (approx. 16–26 bp duplexes) [40]	— *Eif2ak2*-deficient (PKR knock-out) mice: viable and largely normal when housed conventionally and not challenged [41,42]	— undergoes dimerization and *trans* autophosphorylation to phosphorylate eIF2α, resulting in global translation suppression and stress granule formation — indirectly drives apoptosis by depleting pro-survival proteins and promoting pro-apoptotic factors (ATF4, CHOP) or via direct interaction with FADD [43] — interacts directly with IκB to activate NF-κB driven inflammatory transcription and drives type I IFN via IRF3 [44] — contributes to autoinflammation and the integrated stress response (ISR) when cellular dsRNAs accumulate owing to epigenetic relaxation (e.g. loss of SETDB1, TRIM28/KAP1 and HUSH) or defective RNA degradation pathways (e.g. XRN1 depletion) [45–47] — heterozygous *EIF2AK2* variants have been linked to early-onset generalized dystonia [48–50]
**ZBP1** *(Z-DNA/RNA-binding protein 1; DAI / DLM-1)*	— structure-specific sensor that binds exclusively to Z-RNA (an atypical high-energy, left-handed double-helical conformation of dsRNA with a zigzagging sugar-phosphate backbone) via its N-terminal Z-α domains, without sequence dependence [51] — viral ligands include semi-complementary sub-genomic viral RNA products forming panhandles and corkscrews (e.g. during influenza A virus, SARS-CoV-2, HSV-1 and MCMV replication) [52–54] — host-derived ligands include endogenous retroelements (upregulated during nuclear/cellular stress), telomeric RNAs and mitochondrial DNA [6,54,55]	— used extensively in *in vivo* murine viral infection models (such as HSV-1 and Influenza A virus) to characterize cell death pathways	— uses its RHIM domains to engage RIPK1 and RIPK3, recruiting FADD and activating caspase-8 to induce apoptosis, or phosphorylating MLKL to trigger pore formation and necroptosis [56,57] — promotes inflammasome formation to drive pyroptosis — acts as an apical driver of ‘PANoptosis’ (crosstalk between apoptosis, necroptosis and pyroptosis) and acts as a sentinel of cellular stress, where inflammation arises primarily owing to lytic cell death
**OAS—RNase L pathway** *(OAS1, OAS2, OAS3, OASL and RNASEL)*	— OAS proteins sense dsRNA with distinct, length-dependent preferences per family member: OAS1 binds shorter dsRNA (approx. 18 bp); OAS2 requires a minimum of 35 bp; OAS3 binds longer 40–60 bp dsRNA with higher avidity [58–60] — activating ligands include viral replication intermediates (e.g. from flaviviruses, picornaviruses and coronaviruses/SARS-CoV-2) targeted via specific intracellular or Golgi membrane localizations [61]	— *Rnasel*-deficient (RNase L knock-out) mice: viable and normal when unchallenged [42] — mouse genetics is highly complex owing to expanded paralogues—featuring eight paralogues of *Oas1* (*Oas1a-1h*), *Oas2* and *Oas3* and two *OasL* genes (*OasL1* and *OasL2*)—confounding direct comparisons to humans — cell lines/models expressing a germline gain-of-function variant in OAS1 (A76V) — combined multi-knock-out models (e.g. OAS1p.A76V SKIV2L^-/-^ cells lacking RNaseL, MAVS or RIG-I) to map downstream signalling [62]	— upon binding dsRNA, active OAS domains oligomerize and use ATP into 2′–5′-oligoadenylates (2–5A) secondary messengers, which tightly bind and activate latent RNase L [61,63] — active RNase L dimerizes to degrade viral and cellular RNAs, resulting in translational arrest and viral restriction [61] — cleavage products generated by RNase L can further activate other cytosolic sensors to amplify inflammation, and 2–5A can transfer to adjacent cells via gap junctions [59,64] — germline gain-of-function mutations in human OAS1 (such as A76V) cause an autoinflammatory immunodeficiency via ligand-independent activation of the pathway [65]
**endosomal toll-like receptors** *(TLR3, TLR7, TLR8 and TLR13)*	— strictly sequestered within endosomal compartments to survey lumens for viral genomes or cellular debris from dead cells — TLR3 directly senses dsRNA [66] — TLR7 and TLR8 recognize ssRNA (relying on endosomal ribonucleases to process ssRNA into bioactive degradation fragments rather than direct binding) [67,68] — exogenous or endosome-internalized exosomal circular RNA (*circ-CBLB*) can engage TLR3 — TLR13 (mouse-specific) uniquely recognizes bacterial 23S ribosomal RNA [69]	— mice express an additional endosomal nucleic acid sensor, TLR13 [69] — models assessing germline variants or mutations in human *TLR7* or its endosomal trafficking chaperone *UNC93B1* [70]	— initiate rapid transcriptional programs to induce inflammatory genes and coordinate a systemic immune response [71] — strictly sequestered to prevent recognition of ‘self’ RNA; if sequestration fails or variants occur, they trigger systemic autoimmunity — germline variants are reported in patients with autoimmune/autoinflammatory conditions, including SLE, Aicardi-Goutières syndrome, Singleton-Merten syndrome and paediatric neuroinflammatory syndromes [70,72–74]
**accessory helicases and modulators** *(DHX36, DDX1, DDX21, DDX60 and SKIV2L)*	— *DHX36* binds and unwinds guanine-rich RNA sequences (G-quadruplex / G4 structures) [75] — *DDX1* directly binds long dsRNA [76] — *SKIV2L* (RNA exosome cofactor) unwinds secondary structures to allow exosome 3′–5′ degradation, and clears RIG-I-activating dsRNA ligands downstream of the OAS-RNase L pathway [62,77]	— mouse/cell lines with SKIV2L knock-out (SKIV2LKO) combined with OAS1 A76V mutations to monitor hyperactivation [62]	— *DHX36* promotes stress granule formation to facilitate PKR and RIG-I activation [75] — *DDX1*, *DDX21* and *DHX36* form a cytosolic complex in myeloid and dendritic cells that drives inflammatory transcription via the TLR adaptor protein, TRIF [76] — *DDX60* functions to enhance RIG-I-dependent responses — biallelic mutations in *SKIV2L* cause hyperactivation of RIG-I and lead to trichohepatoenteric syndrome type 2 (THES2), a severe autosomal recessive multisystem disorder characterized by excess type I IFN signalling and immunodeficiency [77,78]

## 3. Cytosolic sensors of RNA

### 3.1. RLRs and other DExD/H-box RNA helicases

The RLR family comprises three members, retinoic acid—inducible gene I (RIG-I, encoded by *DDX58*), melanoma differentiation—associated protein 5 (MDA5, encoded by *IFIH1*) and Laboratory of Genetics and Physiology 2 (LGP2, encoded by *DHX58*). All family members have a central DExD/H box RNA helicase motif which, with their carboxy-terminal domains, is responsible for RNA recognition. Upon ligand engagement, MDA5 forms length-dependent filaments on dsRNA, whereas RIG-I recognizes 5′-triphosphorylated dsRNA ends. Binding to dsRNA induces conformational changes that expose and multimerize their N-terminal caspase activation and recruitment domains (CARDs), enabling homotypic CARD–CARD interactions with mitochondrial antiviral signalling protein (MAVS) [19]. MAVS oligomerizes into a prion-like filament on the mitochondria that serves as a signalling platform for the activation of TANK-binding kinase 1 (TBK1) and IκB kinase-ε (IKKε), which activate IFN regulatory factor 3 (IRF3) and IRF7 and nuclear factor kappa B (NF-κB), respectively. IRF3, IRF7 and NF-κB translocate to the nucleus and induce the expression of type I IFN subtypes and inflammatory genes [10,19]. LGP2 lacks CARDs and independent signal-transducing activity; instead, it modulates RIG-I and MDA5 activation [34].

RIG-I surveys the 5′ end of RNA. Extensive studies have identified that RIG-I preferentially binds to double-stranded RNA (dsRNA) molecules bearing a 5′ triphosphate or diphosphate group [11,12], with the most immunogenic dsRNAs forming a blunt end [13,14]. Biochemical studies indicate the minimal requirement for RIG-I sensing is dsRNA that is blunt-ended, while the length of the dsRNA is less critical. 5′ppp and 5′pp dsRNA are commonly produced during replication of dsRNA and single-stranded RNA (ssRNA) viruses. RIG-I is critical to activation of the innate immune response to RNA viruses; RIG-I-deficient mice are highly susceptible to influenza A/B, paramyxoviruses, vesicular stomatitis virus (VSV), respiratory syncytial virus (RSV) and various flaviviruses (reviewed in [79]). However, nascent mRNA and some RNA polymerase III transcripts can also bear a 5′ppp end that must be shielded by RNA-binding proteins (RBPs; e.g. 7SL RNA shielding by SRP9/14 [80]) or dephosphorylated from a tri- to a mono-phosphate (e.g. by DUSP11 [81]) to prevent detection. RIG-I is able to further discriminate immunogenic dsRNAs by steric exclusion of RNAs from its binding pocket which possess 2′-O-methylation at the 5′-terminal nucleotide [82]. Host mRNAs acquire a cap containing 2′-O-methylation in the nucleus following transcription, before export to the cytoplasm, thereby rendering them non-stimulatory to RIG-I.

MDA5**-**activating dsRNA features are not yet fully defined outside of a preference for long double-stranded RNA. Initial biochemical and structural studies using the synthetic ligand, poly(I:C), established MDA5 as a sensor of long dsRNA (approx. 0.5–7 kb). MDA5 binds dsRNA in a sequence-independent manner and assembles into filaments that are particularly stable on long RNA duplexes [83]. In cells, this length dependence is demonstrated by preferential activation of MDA5 by high molecular weight poly(I:C), whereas low molecular weight poly(I:C) activates RIG-I [15]. These studies have been supported by biochemical and cryo-electron microscopy analysis of filament formation of MDA5 complexed with dsRNA, which, for cellular RNA species, includes those derived from inverted *Alu* elements [83,84]. These findings have supported the prevailing model that MDA5 detects long dsRNA generated during viral replication. However, this model does not fully account for poly(I:C) being heterogeneous in length and containing single-stranded overhangs capable of forming higher-order or branched RNA structures, raising the possibility that dsRNA complexity rather than duplex length alone contributes to MDA5 activation.

Studies of virus infection further complicate our understanding of MDA5 ligand sensing. MDA5 is essential for type I IFN induction following infection with picornaviruses such as encephalomyocarditis virus (EMCV) [24,25]. MDA5 is also triggered by viruses with dsRNA or dsDNA genomes. Although dsRNA is a typical replication intermediate across diverse viral families, fractionation and RNA-purification studies suggest that MDA5 agonists are not strictly limited to viral dsRNA duplexes. Instead, MDA5-stimulatory activity maps to high-molecular-weight RNA fractions containing both single- and double-stranded RNA, and in some cases derives predominantly from one strand of viral RNA rather than from annealed duplexes [25]. Furthermore, a recent preprint found that upon infection with SARS-CoV-2 or encephalomyocarditis virus, MDA5 bound primarily to endogenous unspliced transcripts [85]. Taken with observations made with poly(I:C), these studies suggest that more complex dsRNA motifs or higher-order structures, shaped by viral replication, splicing and RNA-binding proteins, and host cofactors such as LGP2, may define the cellular-derived ligands sensed by MDA5. Further studies defining these ligands with precision remain an open question in the field.

In addition to the canonical RIG-I-like receptors, several DExD/H-box RNA helicases act as accessory sensors or signalling modulators that enhance RNA-sensing in a cell-type-specific manner. For example, DHX36 unwinds guanine-rich RNA sequences (G-quadruplex (G4) structures) and facilitates PKR and RIG-I activation by promoting formation of stress granules [75]. DDX1 directly binds long dsRNA and, together with DDX21 and DHX36, forms a cytosolic complex in myeloid cells and dendritic cells that drives inflammatory gene transcription via the toll-like receptor adaptor protein, TRIF [76]. DDX60 has been reported to enhance RIG-I-dependent responses, although its direct RNA-binding activity has not been clearly elucidated. Collectively, these helicases do not function as stand-alone pattern recognition receptors themselves, but act as structure- and localization-sensitive cofactors that stabilize RNA–sensor interactions, scaffold signalling complexes to tune activation according to dsRNA topology and cellular context. These contrast with other DExD/H-box RNA helicases, such as DDX3X and DHX9, which unwind dsRNA and prevent sensing (discussed below).

### 3.2. Other cytosolic dsRNA sensors

**Protein kinase R (PKR)** is a serine/threonine kinase that senses short dsRNA duplexes (>33 bp). Constitutively expressed at low basal levels, PKR expression is upregulated by type I IFN signalling. Structural analysis reveals three domains: two N-terminal double-stranded RNA-binding domains (dsRBD1 and dsRBD2) connected via a flexible linker to a C-terminal kinase domain. In the resting state, PKR exists as an inactive, unphosphorylated monomer. Upon dsRNA binding, PKR dimerizes and trans-autophosphorylates, activating its kinase domain [86]. Active PKR dimers then phosphorylate the alpha subunit of the eukaryotic translation initiation factor 2 (eIF2α), resulting in the suppression of global protein translation. Translation blockade results in the rapid formation of stress granules. Stress granules are membrane-less organelles composed of mRNA and proteins and act as platforms for initiation of innate immunity (reviewed in [87]). In an infection setting, inhibition of protein translation is beneficial as it blocks viral replication while preserving host RNAs within the stress granules until the cell returns to homeostasis. PKR also directly interacts with IκB to unleash NFκB driven inflammatory gene transcription [44] and drives type I IFN expression via IRF3. PKR-mediated translational inhibition can indirectly drive apoptosis by depleting short-lived pro-survival proteins and promoting the preferential expression of pro-apoptotic factors, including ATF4 and CHOP [43]. Furthermore, PKR can directly induce apoptosis through interaction with the Fas-associated death domain protein (FADD).

Like RLRs, PKR can be triggered by dsRNA generated from a variety of viral genomes, including ssRNA, dsRNA and dsDNA viruses (reviewed in [88]). Moreover, endogenous RNAs reported to activate PKR include non-coding retrotransposons such as *Alu* elements, mitochondrial RNAs [38], small nucleolar RNAs [39] and transfer RNAs, all of which can form compact dsRNA structures. To date, a defining feature that discriminates immunogenic from non-activating RNA ligands for PKR remains unclear, but both murine and human genetics have established clear interactions between other dsRNA-binding proteins, including MDA5 and the dsRNA-modifying enzyme ADAR1 [89,90]. This suggests that there may be dsRNA substrates that have features that attract multiple dsRNA sensors, and how these interact and compete for substrates may be important in both a viral and homeostatic response.

PKR recognition of dsRNA is length- and structure-dependent but considered sequence-independent. It requires contact with 2′-hydroxyl groups within the minor groove of A-form RNA for binding [91] and can tolerate mismatches and imperfect duplexes, including bulges that can be structurally straightened upon binding [92,93]. Early biochemical studies defined 33 base pairs as the minimal dsRNA length required for activation, with maximal activation observed for duplexes ≥79 base pairs [94], consistent with a model in which a single dsRNA scaffold promotes PKR dimerization. Rather than binding statically, PKR dynamically scans along dsRNA to interact and dimerize with adjacent PKR molecules, driving activation [95]. Single-stranded RNA elements can further modulate PKR activation; dsRNAs shorter than 33 base pairs can bind to PKR if flanked by single-stranded regions of 10–15 nucleotides [92], and a 5′-triphosphate moiety can strongly enhance PKR activation by RNAs with limited secondary structures [37].

**ZBP1** (Z-DNA/RNA–binding protein 1; also known as DAI or DLM-1) is a cytosolic nucleic acid sensor that couples recognition of aberrant dsRNAs with cell death. ZBP1 contains two N-terminal Zα domains, two receptor-interacting serine/threonine protein (RIP) homotypic interaction motifs (RHIM1 and RHIM2) and a C-terminal signalling domain that is crucial for signal transduction. ZBP1 binds Z-RNA, an atypical left-handed double-helical dsRNA conformation with a zigzagging sugar-phosphate backbone that differs from standard right-handed A-form RNA. The Zα domains allow ZBP1 to bind to Z-RNA in a structure-specific manner, without sequence dependence or base-specific contacts [51,52]. ADAR1p150 is the only other mammalian protein to contain a Zα domain, again highlighting that these pathways are overlapping and intersect at multiple levels. Like ADAR1p150, ZBP1 is induced by type I IFN-induced transcription. Upon binding Z-RNA, ZBP1 C-terminal RHIM domains engage the kinases RIPK1 and RIPK3 [53]. The RIPK1 and RIPK3 complex acts as a scaffold to recruit FADD and activate caspase-8, leading to apoptosis. While in parallel, RIPK3 phosphorylates mixed lineage kinase domain-like pseudokinase (MLKL), which forms pores in plasma membranes, leading to the release of cellular contents [52–54,56]. Activation of these pathways results in an immunogenic form of regulated cell death termed necroptosis.

ZBP1 has also been suggested to induce another form of programmed cell death known as pyroptosis by promoting the formation of inflammasomes. This and other studies on the extensive crosstalk between pyroptosis, apoptosis and necroptosis led to the establishment of the concept of ‘PANoptosis’ [96]. In PANoptosis, the PANoptosome provides a molecular scaffold that allows for interactions of the machinery required for all three cell death pathways, and ZBP1 has been proposed as the apical driver of PANoptosome formation. Although early studies suggested that ZBP1 may activate inflammatory signalling pathways such as NF-κB or TBK1 [97], this remains controversial and may reflect historical misassignment of ZBP1 as a DNA sensor prior to the discovery of cGAS [98,99]. Current evidence supports the view that ZBP1-driven inflammation arises primarily as a consequence of lytic cell death rather than direct cytokine signalling [56].

Z-RNAs can be formed during viral replication of both RNA and DNA viruses. Formation of Z-RNAs during viral replication is best understood in the context of the ssRNA virus, Influenza A virus (IAV). During replication of IAV, the viral polymerase falls off its template RNAs but re-engages further downstream along the same RNA strands, producing sub-genomic RNAs with intact 5’ and 3’ ends but harbouring large internal deletions. The 5′ and 3′ ends of IAV gene segments are semi-complementary and allow the formation of double-stranded structures. If these defective viral genome products form right-handed duplexes, they become ligands for RLRs, while the left-handed double-helical conformation results in ZBP1 triggering Z-RNAs [52]. To date, ZBP1 has been reported to be activated by IAV, SARS-CoV-2, herpes simplex virus-1 and murine cytomegalovirus [52,54,100,101]. Interestingly, a recent study reports that in both herpes simplex virus 1 and IAV infection, host cell-encoded Z-RNAs, rather than viral Z-RNAs, are the primary ZBP1-activating ligands [6]. These host-derived Z-RNAs are formed from endogenous retroelements, and their expression is increased in response to nuclear stress caused by viral infection [6]. Indeed, cellular stress is emerging as a driver of Z-RNA formation, predominantly from endogenous retroelements [57]; however, other reports have also indicated that telomeric RNAs and mitochondrial DNA can activate ZBP1 [55,102]. Together, these findings suggest that ZBP1 functions as a sentinel of cellular stress, poised to trigger cell death when nucleic acid homeostasis is disrupted.

**OAS**-**RNaseL** is a cytosolic RNA-sensing pathway that couples detection of dsRNA to rapid degradation of cellular and viral RNA [65]. Upon binding to dsRNA, OAS (oligoadenylate synthetase) proteins synthesize unique secondary messengers that feature 2′–5′ phosphodiester bonds by oligomerizing ATP into 2′–5′-oligoadenylates (2–5A). The human OAS family comprises four members: OAS1, OAS2, OAS3 and OAS-like protein (OASL) [65]. OAS proteins are composed of a structurally conserved OAS domain, with OAS1 containing a single OAS domain, OAS2 having two units and OAS3 containing three. Notably, only a single OAS domain in OAS2 and OAS3 has retained the catalytic triad required to generate 2–5A [103]. OASL is catalytically inactive and has been suggested to enhance RIG-I signalling [58]. Adding to the complexity of the family, all members except OAS3 have multiple splice variants which diversify OAS subcellular localization and RNA-sensing. The specific functions of the OAS proteins are more complicated to delineate in mice, where, in place of the four proteins in humans (*OAS1, OAS2, OAS3* and *OASL*), there are eight paralogues of *Oas1* (*Oas1a*-*1h*), *Oas2*, *Oas3* and two *OasL* genes (*OasL1* and *OasL2*), which confounds direct comparison [104]. As for RIG-I family members, OAS proteins are themselves IFN-stimulated genes (ISGs).

The 2–5A chains produced by OASs contain at least three adenosine monomers which tightly bind to and activate the latent ribonuclease L (RNaseL) [63]. 2–5A binding licenses RNaseL to dimerize and degrade both viral and cellular RNAs, resulting in translational arrest and viral restriction [61]. To further prime cells to combat viral infection, 2–5A can be transferred to neighbouring cells via connexin gap junctions [59], and cleavage products generated by RNaseL can activate other cytosolic RNA sensors to enhance the inflammatory response [64].

Structural and biochemical investigations have revealed that OAS family members have a preference for different dsRNA lengths. OAS1, being the smallest family member, interacts with shorter dsRNA, approximately 18 base pairs (bp) [60]; OAS2 requires a minimum of 35 bp dsRNA for activation; and the largest family member, OAS3, binds to longer, 40–60 bp lengths of dsRNA [60]. These specificities likely reflect the minimal lengths required to accommodate binding of OAS domains of each family member; OAS3, having three OAS domains, binds dsRNA with higher avidity than OAS1 but requires a long dsRNA molecule for stable interaction [60,105,106]. Reminiscent of other RNA sensors, there is evidence to suggest that multimerization of OASs on longer dsRNAs enhances activity [60], and a recent report indicates that active OAS2 is dimerized [107].

Subcellular localization allows OAS proteins to detect a broad range of viral replication intermediates while limiting inappropriate activation by endogenous RNA. For example, variants which alter the C-terminal tail of OAS1 allow OAS1 to be targeted to intracellular membranes, including endomembranes formed by viruses. This proximity to viral replication increases the antiviral activity of OAS1 against certain RNA viruses, including flaviviruses, picornaviruses and SARS-CoV-2 [108]. Similarly, a recent report demonstrated dimerization and myristoylation of OAS2 localize it to the Golgi membranes and that this is required for OAS2 activation and the restriction of coronaviruses [107].

## 4. Endosomal RNA sensors: toll-like receptors

In addition to the cytoplasmic sensors described above, the TLRs function as endosomal RNA sensors. The human TLR family includes three specialized members dedicated to the detection of nucleic acids: TLR3, which senses dsRNA [66], and TLR7 and TLR8, which recognize ssRNA [67,68]. Mice express an additional endosomal sensor, TLR13, which specifically recognizes bacterial 23S ribosomal RNA [69]. To prevent the recognition of ‘self’ RNA and avoid systemic autoimmunity, TLR3, TLR7 and TLR8 are sequestered within endosomal compartments (figure 2). The trafficking and stability of these receptors are governed by Unc-93 homologue B1 (UNC93B1). UNC93B1 physically associates with the transmembrane domains of TLR3, 7 and 8, facilitating their packaging into COPII vesicles for anterograde transport to the endolysosomal network [109]. This strict compartmentalization provides a controlled environment where acidic pH and resident nucleases can process RNA ligands for recognition, while simultaneously preventing these TLRs from encountering host RNAs in the cytosol. This localization allows them to survey the lumen of endosomes and lysosomes for aberrant RNA derived from viral genomes or debris from dead cells. Upon ligand engagement, these receptors initiate rapid transcriptional programmes to induce inflammatory genes and orchestrate a systemic immune response. This robust activity is largely driven by the high expression of endosomal TLRs in dendritic cell subsets, which serve as critical sentinels of the innate immune system [67,110,111].

**Figure 2.**
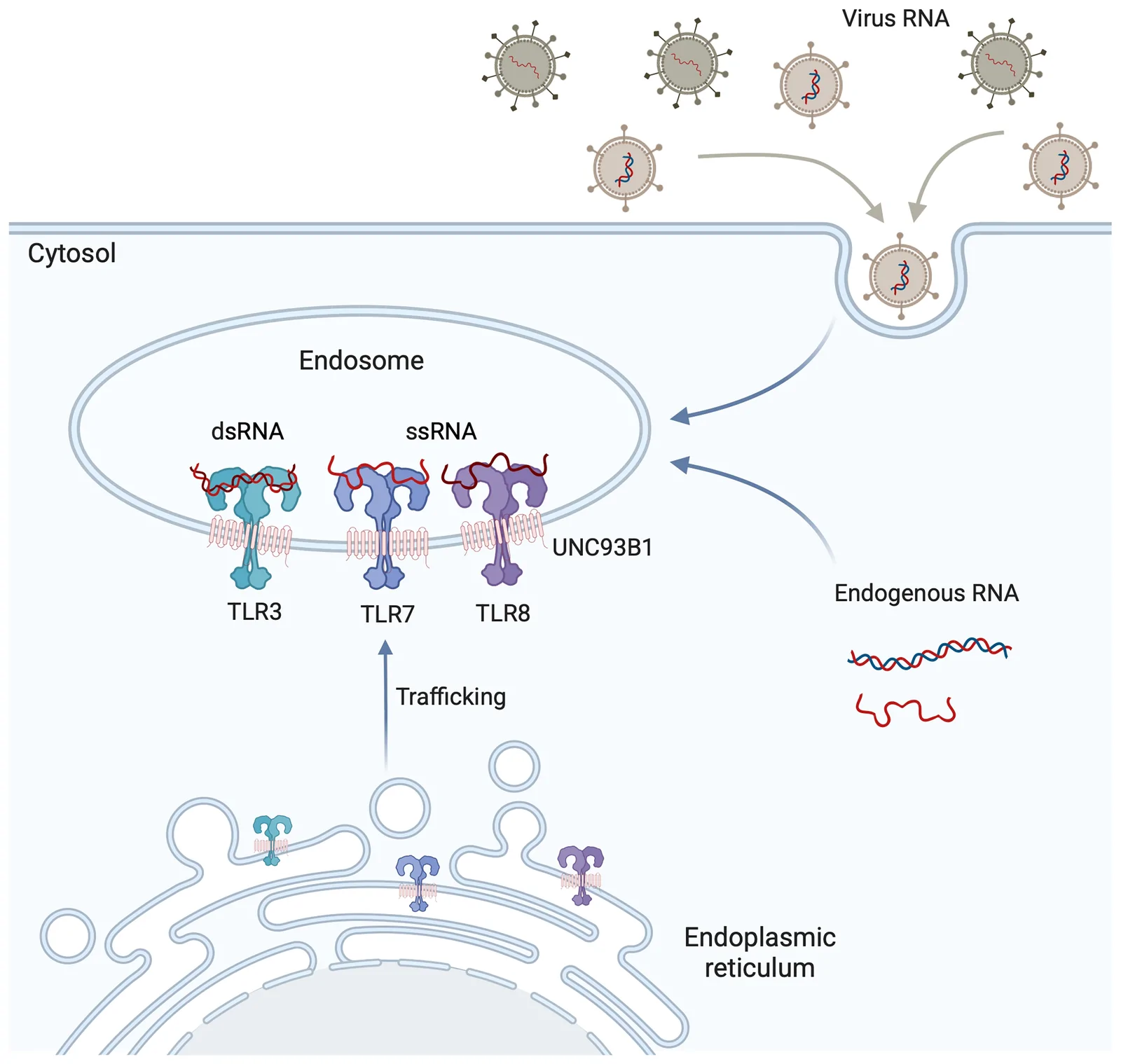
Endosomal TLRs recognize distinct structural features of RNA. Nucleic acid-sensing TLRs function within the endosome to detect RNAs from various sources. To differentiate targets and initiate an appropriate immune response, these receptors exhibit specific structural preferences. TLR3 is activated by dsRNA, while TLR7 and TLR8 specifically bind to ssRNA. The endosomal compartment acts as a central hub for ligand presentation, capturing extracellular viral RNA via endocytosis as well as host-derived endogenous RNA. To properly position these sensors for ligand detection, TLR3, TLR7 and TLR8 are transported from the endoplasmic reticulum to the endosome.

**TLR3** senses dsRNA [66]. Observations made in TLR3-defective settings, including gene-deficient mice and loss-of-function gene variants in humans, demonstrated that TLR3 mediates a protective response against positive-strand RNA viruses, including coxsackie virus group B serotype 3, encephalomyocarditis virus and poliovirus [112] and dsDNA viruses such as herpes simplex virus-1 [113]. Like other members of the TLR family, TLR3 is a type I transmembrane protein composed of a large N-terminal ectodomain containing 23 leucine-rich repeats (LRRs) that form a horseshoe-shaped solenoid, a single transmembrane helix, and a C-terminal cytosolic toll/interleukin-1 receptor (TIR) domain. Ligand engagement occurs at two distinct sites on the lateral surface of the ectodomain, requiring a dsRNA duplex of at least 40–45 base pairs to bridge two TLR3 monomers into a signalling-active dimer [114]. TLR3 also uses the presence of modified nucleotides including N-methyl-adenosine and 2-thiouridine to discriminate between ‘self’ and ‘non-self’ dsRNAs [115]. Unlike other members of the TLR family, TLR3 signals exclusively through the toll/IL-1R domain-containing adaptor-inducing IFN-B (TRIF) protein. Upon receptor dimerization, the TIR domains of TLR3 recruit TRIF, which acts as a scaffold for two divergent signalling branches. The first branch involves the recruitment of TRAF3 and the subsequent activation of the TBK1/IKKe kinase complex, which phosphorylates the transcription factor IRF3 to drive the expression of type I IFN. Simultaneously, TRIF interacts with TRAF6 and RIPK1 to activate the TAK1 complex, which triggers the NFkB and MAPK pathways to induce the production of pro-inflammatory cytokines such as IL-6 and TNF.

**TLR7 and TLR8** result from a gene duplication [116] and recognize ssRNAs, as well as synthetic tricyclic derivatives such as resiquimod [67,68]. TLR7 and eight sense GU-rich and AU-rich ssRNA sequences prevalent in the genomes of ssRNA viruses such as influenza, HIV-1, vesicular stomatitis virus, Sendai virus, flaviviruses and coronaviruses [68,117–120]. The relative importance of TLR7 and 8 to the host response to specific viruses is in part owing to differential expression patterns, with TLR8 being highly expressed in monocytes, conventional dendritic cells and neutrophils, while TLR7 is predominantly expressed in plasmacytoid dendritic cells and B cells [121].

In contrast to cytosolic RNA sensors, TLR7 and TLR8 rely on endosomal ribonucleases to process ssRNA into bioactive degradation products. Both receptors contain two distinct ligand-binding sites that recognize different RNA-derived moieties. Although structurally conserved between the two receptors, subtle differences in pocket size and electrostatic potential give this site ligand preference, with TLR7 favouring guanosine [122] and TLR8 preferring uridine binding [123,124]. A substantial portion of the ligand-induced dimerization interface is formed by this first site, where bound guanosine or uridine occupies the centre of the interface and functions as a molecular glue stabilizing the active TLR7 or TLR8 homodimer [122,124]. The second ligand-binding site interacts with short RNA fragments consisting of two or more ribonucleotides linked by phosphodiester bonds known as oligoribonucleotides. Unlike the conserved first site, the second sites of TLR7 and TLR8 are spatially distinct and structurally divergent. Structural and biochemical analyses indicate that TLR7 preferentially recognizes a uridine-rich 3-mer motif, exemplified by a UUU sequence within longer poly-uridine ssRNA [122]. The central uridine (U2) is stringently recognized through base-specific hydrogen bonding and RNA-specific 5′-hydroxyl recognition, while flanking nucleotides are more permissive. Thus, the minimal requirement for productive TLR7 engagement at the second site is a 3-mer oligoribonucleotide containing a central uridine. By contrast, the TLR8 second site accommodates shorter oligoribonucleotides, likely as small as dinucleotides (e.g. UG), with relatively low base specificity [123]. Following ligand engagement, TLR7 and TLR8 recruit the adaptor MyD88, leading to the assembly of the Myddosome, a large oligomeric complex comprising MyD88 and kinases of the IRAK family. In most myeloid cells, this complex triggers TRAF6-mediated activation of the TAK1 complex, driving NFkB and MAPK pathways for a robust inflammatory response. However, in specialized plasmacytoid dendritic cells, TLR7-driven activation of the Myddosome directly associates with TRAF3 to phosphorylate IRF7 to drive production of type I IFNs [125].

## 5. Avoiding self-detection: hiding cellular RNA from the cytoplasmic sensors

Rare genetic variants and knockout studies highlight sensing of endogenous nucleic acids as a pathogenic driver, but what is the source of the immune-stimulating dsRNA? Nearly half of the genome of humans and other mammals is made up of transposable elements (TEs) [126]. These are repetitive DNA sequences that can move within the genome and are broadly divided into two major classes: DNA transposons, which mobilize via a ‘cut-and-paste’ mechanism, and retrotransposons, which mobilize through an RNA intermediate. Retrotransposons are the predominant type in mammalian genomes and can be further subdivided into long terminal repeat (LTR) retrotransposons, which include endogenous retroviruses (ERVs) derived from ancient viral integrations in germ cells, and non-LTR retrotransposons, which encompass long interspersed nuclear elements (LINEs) and short interspersed nuclear elements (SINEs). LINEs, such as LINE-1 (L1), encode proteins required for their own reverse transcription and integration, while SINEs, such as *Alu* elements, are non-autonomous and rely on LINE machinery for mobilization. Silencing of these elements is crucial to protect genome integrity from insertional mutagenesis. In addition, the transcription of TEs into RNA, particularly tandem inverted repeats (IRs), forms long, stable dsRNA that can activate various dsRNA sensors. Furthermore, mitochondria represent an additional source of potentially immunostimulatory cellular dsRNA [9]. Although normally compartmentalized away from cytosolic receptors, mitochondrial dsRNA can escape under conditions of stress or organelle damage, triggering autoinflammatory responses.

To prevent toxic self-detection, cells deploy a multi-layered, spatially and temporally coordinated defence mechanism: epigenetic repression in the nucleus, co-transcriptional processing and modification, regulated nuclear retention and export, and cytoplasmic shielding coupled with decay. Through these specific checkpoints across the RNA lifecycle, endogenous dsRNA's are systematically neutralized before reaching cytosolic pattern recognition receptors (figure 3). In the following sections, we discuss each of these regulatory layers in detail.

**Figure 3.**
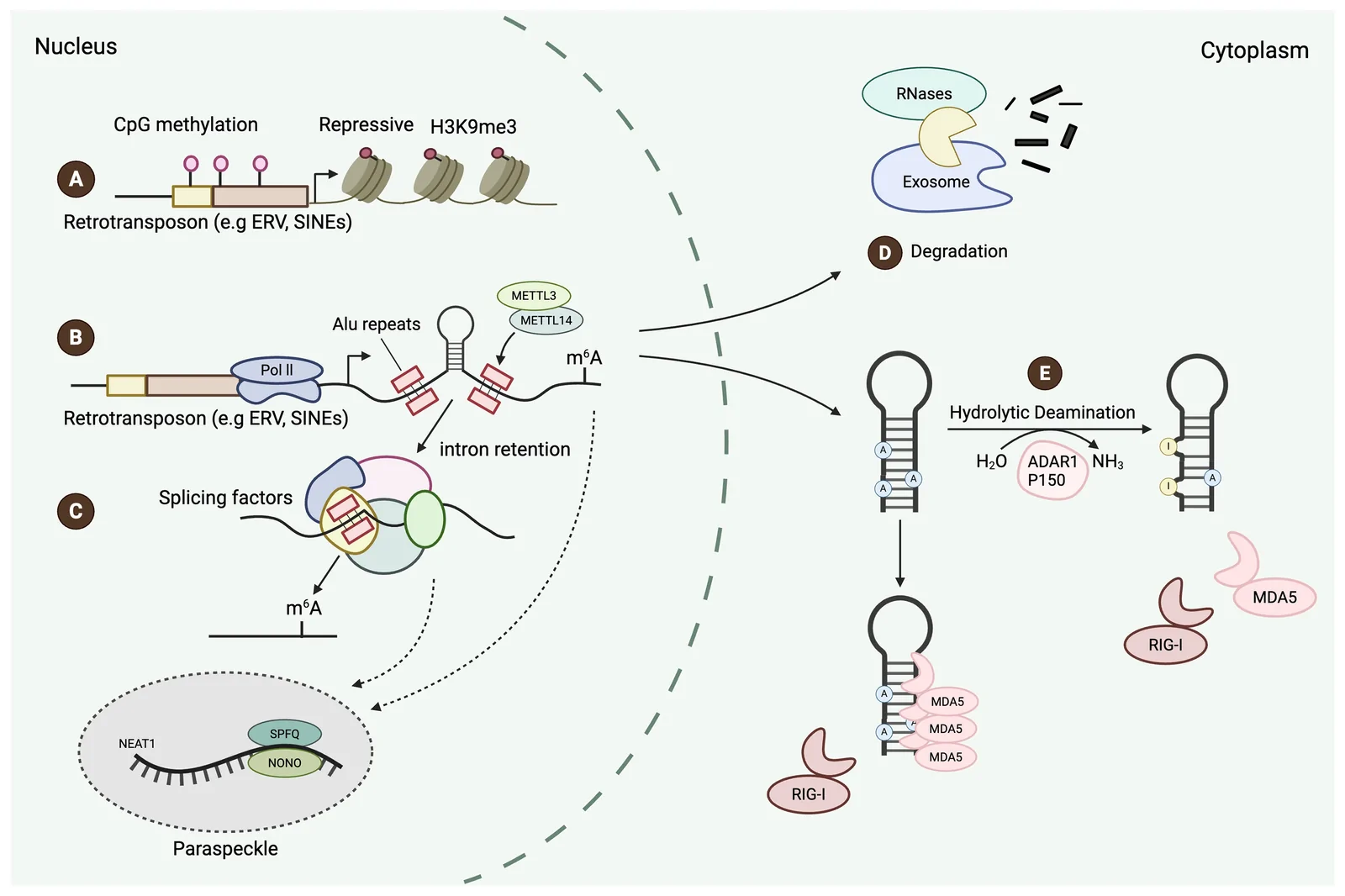
Mechanisms to prevent sensing self dsRNA. Cells have evolved a range of methods to prevent or reduce the immunogenicity and innate immune sensing of cellular-derived dsRNAs. A. Epigenetic mechanisms—methylation and silencing of expression—provide a first line that can act to prevent the formation of dsRNA. B. Once the RNA is expressed and double-stranded structures form, RNA modifications can act to alter this structure and mark these as cellular RNAs that should not trigger the cytoplasmic innate immune system. In the case of ADAR1p150, RNA modifications can also be applied after export from the nucleus. C. RNA processing, including splicing and circRNA formation, can act to reduce the dsRNA structures within cellular transcripts that transition from the nucleus to the cytoplasm. Additional nuclear surveillance mechanisms are active, such as sequestration into paraspeckles in the nucleus, which can prevent cellular dsRNA from being exported from the nucleus. If the dsRNA feature can be modified or removed prior to transport to the cytoplasm, this prevents exposure to the cytoplasmic RNA sensing proteins. D. Degradation of the dsRNA can remove it from the cytoplasm, thus preventing sensing. E. The dsRNA regions of cellular transcripts can be permanently modified by the A-to-I editing-dependent activity of ADAR1p150. Additionally, the RNA-binding activity, independent of catalytic A-to-I editing, of ADAR1p150 can limit access to the cellular transcript by other RNA sensor proteins and so tune the threshold for innate immune activation.

## 6. Avoiding self-detection: epigenetic silencing

Epigenetic silencing and transcriptional control serve as the primary defence against immunogenic, nuclear-derived dsRNA by suppressing its expression at the source; unexpressed transcripts require no downstream tolerance mechanisms. In somatic cells, repressive chromatin anchored by DNA methylation and SETDB1-deposited H3K9me3 is maintained by TRIM28/KAP1 and the HUSH complex (TASOR, MPP8 and Periphilin), keeping ERVs and repetitive elements transcriptionally silenced [127]. Genetic perturbations of these factors consistently derepress ERV loci, elevate bidirectional transcription and induce ISGs in an MDA5-dependent manner or cause translation arrest via PKR activation. SETDB1 knock-out or depletion causes widespread loss of H3K9me3 at ERV loci and dsRNA accumulation detectable by J2 antibody staining [45], while loss of TRIM28/KAP1 [128] or HUSH components yields similar activation of ERV transcripts and downstream innate immune signalling [129]. These studies establish that chromatin repression is both necessary and sufficient to restrain dsRNA levels at source loci, and that innate immune activation is a consequence of their de-repression. Evolutionary analysis supports the epigenetic silencing of repeats as a primary means to prevent expression of potentially immunogenic dsRNAs. For more recent evolutionarily acquired repeats, cells use active mechanisms such as RNA modification via A-to-I RNA editing, whereas older repeats are more effectively controlled by epigenetic silencing that prevents their expression [130,131].

Pharmacologic inhibition, particularly in the context of cancer cells, provides complementary causal evidence. DNA methyltransferase inhibitors such as azacitidine and decitabine reduce CpG methylation across ERV promoters, increase ERV transcription, and induce ‘viral mimicry’ characterized by accumulation of dsRNA or DNA/RNA hybrids and activation of PRRs; this is attenuated by dsRNA digestion or sensor knock-down, confirming endogenous cellular ligands as the proximal triggers [132]. Histone deacetylase inhibitors and LSD1 inhibitors similarly de-repress repeat elements and promote bidirectional transcription at enhancer and promoter regions, elevating duplex-forming RNA (dsRNA) expression and IFN signalling [133]. Across these interventions, locus-level RNA-seq and ChIP-seq show repeat-enriched transcriptional changes preceding innate immune activation, and genetic or pharmacologic suppression of nucleic acid sensors blunts the ISG response, establishing that epigenetic relaxation drives dsRNA-dependent signalling rather than secondary stress pathways.

Transcriptional termination and processing factors have also recently emerged as important to limit dsRNA formation from pervasive transcription. The integrator complex (INT) targets paused RNA polymerase II (RNAPII) at promoter-proximal regions for transcription termination. This prevents expression of stress- and immune-responsive genes under normal conditions. Acute depletion of INT subunits leads to expression of transcription factors that drive the ISR [46]. In addition, longer-term disruption of INT releases immature RNAPII complexes into genes which are defective for productive elongation. This leads to the premature termination of transcription and the formation of incomplete mRNAs with retained introns containing inverted repeats. These are then exported to the cytoplasm, where they activate the dsRNA sensor PKR and the ISR.

Defects in transcription termination machinery (e.g. CPSF73 and XRN2) promote readthrough transcription into downstream antisense regions, with the potential to increase sense–antisense overlaps and dsRNA formation [134]. Perturbations in this pathway have not yet been linked to dsRNA-sensing but may represent a further layer of regulation. Likewise, alternative polyadenylation (APA) extends the 3′-end of genes and promotes incorporation of IR-*Alus* [135]. In neurons, lengthening of 3′-UTRs is controlled by the ELAVL family of genes and increases dsRNA load and MDA5 activation [136]. Finally, recent work has connected DNA repair and transcriptional regulation to dsRNA-sensing. Ku70/Ku80 (encoded by *XRCC5/XRCC6*), canonical factors in non-homologous end joining, have been shown to restrict innate immune activation by IR-*Alus* [137]. Ku binds primarily to antisense *Alu* transcripts, which prevents activation of multiple dsRNA-sensing pathways, including RIG-I/MDA5, PKR and OAS, although the mechanism is unclear. IFN signalling was reduced by sensor ablation, supporting a direct RNA-mediated mechanism rather than a secondary DNA damage response.

Viewed together, these studies establish that chromatin repression (SETDB1/TRIM28/HUSH, DNA methylation) minimizes transcriptional input from ERVs and repetitive DNA, while transcriptional attenuation and termination suppress pervasive and antisense RNAs that could otherwise anneal into dsRNA. When these safeguards are pharmacologically relaxed or genetically perturbed, dsRNA accumulates and engages MDA5/RIG-I/PKR to drive type I IFN and inflammatory programs. This epigenetic and transcriptional layer operates upstream of post-transcriptional mechanisms, including RNA modifications, splicing and RNA degradation. However, their synergy is evident under viral mimicry conditions: epigenetic drugs elevate ERV and bidirectional transcripts, while other pathways act to buffer the increase in cellular-derived dsRNAs. For example, combined relaxation of epigenetic silencing with impaired RNA editing by ADAR1 leads to pronounced dsRNA accumulation and robust innate activation [127,138]. Coordination of these control mechanisms is therefore essential to restrict dsRNA levels below immunogenic thresholds in homeostasis and to harness or avoid antiviral signalling therapeutically.

## 7. Avoiding self-detection: RNA modifications

Epigenetic silencing is not possible for many elements that reside within non-coding regions (introns, UTRs) of functional protein-coding genes. Once transcribed, the potentially immunogenic dsRNA needs to be actively regulated, including its localization and structure. A prominent active mechanism to facilitate discrimination between cellular-derived RNAs and those that are foreign is through post-transcriptional RNA modification, collectively termed the epitranscriptome [139]. There are >170 RNA modifications catalogued to date, impacting all four RNA bases and using diverse chemical space. RNA modifications can be permanent and, once made, persist for the life of the RNA, or be subject to installation and removal in a dynamic and temporally controlled fashion analogous to readers, writers and erasers of chromatin marks [139]. It is now appreciated that RNA is actively modified across its lifespan, and this has important implications for its function, localization, secondary and tertiary structure, half-life and immunogenicity [139]. tRNAs are heavily modified and will not be discussed in further detail here. RNA modifications occur throughout the length of a cellular RNA, from the 5′ cap structure, the 5′ untranslated region (UTR), coding regions and across the poly-A tail (if the RNA is polyadenylated) and at the 3′ UTR [140]. Common RNA modifications in mammals include N6-methyladenosine (m^6^A), pseudouridine (Ψ), N1-methyladenosine (m^1^A), 5-methylcytosine (m^5^C) and Adenosine-to-inosine (A-to-I) [141]. Modifications to cellular RNAs enable them to be tolerated by the cytoplasmic RNA sensors described above. Understanding how RNA modifications alter immunogenicity has been central to the development and highly successful application of mRNA therapeutics [115]. Modified RNAs are now being used for approaches from vaccination against infectious disease to cellular reprogramming [142], cancer therapy and gene replacement. Not all RNA modifications modulate immunogenicity; current evidence indicates this regulatory function is restricted to a specific subset of epitranscriptomic marks. For example, significantly reducing N4-acetylcytidine (ac4C) in cellular RNA did not appreciably activate cytoplasmic innate immune pathways [143].

One of the most well-characterized RNA modifications that directly intersects with the cytoplasmic nucleic acid sensing system is the conversion of adenosine-to-inosine (A-to-I) in cellular-derived dsRNA, a process termed A-to-I RNA editing. Adenosine deaminase acting on RNA (ADAR) proteins bind to regions of dsRNA structure in cellular-derived RNAs and convert A-to-I, resulting in the permanent change of the targeted base for the lifespan of the RNA. Detailed maps of edited sites are known because inosine is read as guanosine upon generation of sequencing libraries, resulting in A-to-G mismatches from the reference genome in RNA sequencing. While a demonstrated physiologically essential function of A-to-I editing is protein recoding, largely attributable to editing by ADAR2, this type of editing represents a small proportion of the overall editing reported in mammals [144–147]. The vast majority of editing in mammals is in non-coding repetitive elements, in particular primate-specific *Alu* repeats and B1/B2 SINEs in rodents [148,149]. A-to-I editing linked to immune sensing is mediated by ADAR1 [89,90,148–153].

ADAR1 has two distinct isoforms, a p110kDa isoform (ADAR1p110) that is primarily expressed in the nucleus, and a 150kDa isoform (ADAR1p150) that is predominantly found in the cytoplasm. Through detailed genetic studies in both human and mouse, it is now established that A-to-I editing is an essential means that cells use to tolerate cellular dsRNA and that this prevents MDA5 sensing of cellular dsRNA as foreign/non-self [89,147,149,150,154–156]. ADAR1 loss or mutation causes profound innate immune activation in sterile contexts across animal models, specific cell lines and patients carrying pathogenic ADAR variants, driving type I IFN production [90,153]. More specifically, it is the expression and editing activity of the cytoplasmic ADAR1p150 isoform that is critical to preventing MDA5 activation by cellular dsRNA [23,89,155,157–160]. Elegant whole-animal genetic studies in mice, supported by human genetics, have demonstrated that the unedited or under-edited cellular dsRNA that forms following the loss or mutation of ADAR1 is sensed specifically by MDA5 [7,8,23,147,150,155,156,160,161]. This finding constrains the characteristics of the cellular dsRNA that could be the immunogenic RNA species in this setting, because MDA5 has specific preferences for the type and length of the dsRNA that distinguish it from other cytoplasmic innate immune sensors, as discussed above [23,83,84]. The current model of how ADAR1p150 impacts cellular dsRNA is that editing of regions containing longer regions of dsRNA, often formed by inverted repeat elements such as *Alu* or potentially *cis*-naturally occurring antisense transcripts (*cis*-NATs), introduces base mismatches that destabilize the secondary structure or introduce regions of less well-paired structure [90,157]. This means that MDA5 cannot oligomerize along the dsRNA, resulting in tolerance or ‘immunogenic silencing’. These studies have established a critical role of ADAR1p150-mediated A-to-I RNA editing in actively marking cellular dsRNA as ‘self ’, and that this prevents aberrant recognition of these RNAs by MDA5. This also demonstrates that cellular dsRNA is potently immunogenic when unmodified, and that the cell must employ active measures and mechanisms to prevent the levels of transcribed cellular dsRNA from breaching the tolerance threshold. Understanding the landscape of the cellular participants and mechanisms used in achieving self-tolerance will be highly informative for developing means to toggle this process, both positively and negatively, for therapeutic benefit in a variety of contexts [90,152,162]. In addition to suppressing MDA5, ADAR1 protein also has a critical role in preventing activation of other sensors, including PKR, ZBP1 and OAS, independent of its RNA editing activity [89,154,163,164]. In these cases, suppression is dependent on the RNA-binding domain (PKR/OAS) or the Z-RNA-binding domain (ZBP1) of ADAR1, rather than the RNA editing activity of the protein, since expression of a catalytic mutant is sufficient to dampen activation of the sensors. This intimates more complex interconnections and a hierarchical model, where the sum of the RNA editing activity and RNA binding/sequestration of ADAR1p150 acts as a key set point of engagement of cellular dsRNAs by a range of innate immune sensor proteins.

Among other RNA modifications, there is evidence that RNA methylation, and m^6^A in particular, is linked to the suppression of immunogenicity of cellular RNA. Using murine models, it was demonstrated that loss of the m^6^A writer METTL3 leads to accumulation of dsRNA and activation of the innate immune sensing system *in vivo* [165]. This resulted in the production of type I IFN and a sterile inflammatory response, indicating a role for METTL3-deposited m^6^A in tolerance of cellular RNAs. Independently and using an orthogonal approach, small molecule inhibitors of METTL3 reduced m^6^A content of human cellular RNAs and, in parallel, activated the cytoplasmic RNA-sensing response in sterile settings [166]. Unlike the ADAR1-dsRNA-MDA5 axis, the specific nature and type of RNA that becomes immunogenic during METTL3 inhibition or loss is less clear. Inhibition of expression of MDA5, RIG-I, PKR and RNaseL individually using CRISPRi attenuated, but none specifically prevented, upregulation of cell surface MHC-I that correlates with the type I IFN response [166]. Indeed, inhibition of all four sensors concurrently did not completely prevent MHC-I upregulation, suggesting a more complex interplay of small molecule inhibitor-mediated effects on METTL3-mediated m^6^A and cytoplasmic RNA-sensing. Not all studies assessing the effects of reduction in METTL3 activity or expression have reported this link to RNA-sensing pathways [167], suggesting a distinct relationship to that reported for A-to-I editing and MDA5. Regarding other RNA sensors, very recent work indicates that cellular-derived 2′-*O*-methyl (2′-OMe) guanosine-containing RNA fragments generated from cellular RNAs can act as a natural antagonist pool for TLR7 and TLR8 [168].

These studies demonstrate a key homeostatic role for RNA modifications in allowing cells to tolerate cellular-derived RNAs in the cytoplasm. As more RNA modifications are studied and the effector proteins of them identified, the function of additional RNA modifications and how they contribute to tolerance to self RNAs will become more completely understood. The writers, readers and erasers of these RNA modifications also lend themselves to pharmacological targeting, opening up new means to manipulate the innate immune response across a range of disease contexts.

## 8. Avoiding self-detection: RNA splicing

Another mechanism that alters the cellular RNA landscape is alternative splicing. This process is highly conserved through a coordinated and sequential process in the nucleus; the full-length RNA transcript is modified through removal of intronic regions to yield the mature mRNA [169]. The mRNA is then exported to the cytoplasm to allow translation by the ribosome. The link between splicing and suppression of cytoplasmic dsRNA sensing has become apparent, particularly contributed to studies in oncology.

The analysis of core RNA splicing machinery mutations in cancer has led to the appreciation of the link between ordered RNA splicing and preventing sensing of mis-spliced cellular RNAs in the cytoplasm. The genetic depletion of RNA splicing factors can lead to activation of cytoplasmic dsRNA-sensing pathways [170]. This can also be seen when cells are treated with small molecule inhibitors of SF3B1, a core component of the U2 snRNP RNA splicing machinery [170]. This was proposed to be mediated via intron-retained transcripts where there are sequences that have the capacity to form dsRNA structures, such as *Alu* repeats. While conceptually similar to the cellular-derived RNA substrates proposed to be detoxified by ADAR1 [23], the mechanism that mediates the sensing and the specific sensors involved are less clear and appear to involve multiple RNA-sensing pathways [170]. Genetic screening did not resolve a singular sensor, indicative of an opaque mechanism like that observed with small molecule METTL3 inhibition. Further work will be required to more fully define how splicing inhibition specifically leads to cytoplasmic sensing of cellular dsRNAs.

Perhaps not surprisingly, splicing acts together with RNA modification to suppress cellular RNA immunogenicity. An example of this is the interaction between loss of hnRNPC and ADAR1 [171]. In this instance, loss of hnRNPC results in the use of cryptic splice sites, resulting in retention of introns containing *Alu* repeats that can form dsRNA structures [171]. These become MDA5 substrates in the absence of ADAR1-mediated editing, demonstrating that the combined action of splicing and RNA modification acts in a coordinated manner to prevent immunogenicity of cellular dsRNAs [171]. An analogous mechanism to prevent self-sensing of cellular dsRNA is mediated by hnRNPM. hnRNPM suppresses cryptic splicing that can result from the presence of LINEs within introns. LINEs can harbour pseudo-splice sites, and these should normally be ignored. In the absence of hnRNPM, these splice sites can be used, resulting in the incorporation of the LINEs into the mRNA, enabling dsRNA formation through base-pairing of inverted *Alu* and LINEs. The accumulation of these dsRNA structures in the cytoplasm can result in the activation of cytoplasmic sensing [172]. As described for spliceosome inhibition, the sensor specifically recognizing these cellular transcripts is not clearly resolved, as combined knock-down of RIG-I, MDA5 and TLR-3 attenuated but did not block interferon-stimulated gene (ISG) expression, a transcriptional response directly linked to cytoplasmic sensing.

An atypical form of splicing, resulting in circRNAs, has emerged as a regulator of RNA-sensing. circRNAs are covalently closed RNA molecules generated through back-splicing of precursor mRNAs, in which a downstream splice donor is joined to an upstream splice acceptor. Consequently, circRNAs lack the canonical 5′–3′ polarity and poly(A) tail of linear mRNAs. Although circRNAs are typically expressed at lower steady-state levels than their linear counterparts, their circular topology renders them resistant to exonuclease-mediated degradation, resulting in markedly prolonged half-lives relative to linear messenger RNAs [173]. CircRNAs were first identified in viral genomes in the 1970s and later discovered in eukaryotic cells, but their widespread expression and biological diversity only became apparent at scale with the advent of high-throughput RNA sequencing and computational pipelines capable of detecting back-splice junctions [174]. CircRNAs are produced from the same pre-mRNA as their linear counterparts, allowing a single genetic locus to generate distinct linear and circular RNA isoforms. There has been evidence that A-to-I editing can contribute to the formation of these RNAs, re-enforcing the concept of interplay of RNA-binding proteins in regulating diverse RNAs [175]. These isoforms frequently exhibit independent expression patterns, tissue specificity and biological functions, and circRNA expression is dynamically altered across development and in diverse disease states.

CircRNAs are structurally heterogeneous and can be composed of exons only, introns only or combinations of exonic and intronic sequences; exon-only circRNAs represent approximately 80% of all annotated circRNAs and are predominantly localized to the cytosol (reviewed in [176]). A wide range of functions has been attributed to circRNAs, including roles as microRNA sponges, transcriptional regulators, molecular scaffolds and, in some cases, templates for cap-independent translation (reviewed in [176]). For our interest, circRNAs are emerging as direct interactors of RNA sensors, primarily PKR, but some reports have assessed RIG-I and TLR3. With the notable exception of PKR, there is currently no unified framework describing how circRNAs are recognized by most RNA-sensing PRRs. Seminal work from Ling-Ling Chen and colleagues demonstrated that endogenous circRNAs directly bind PKR and function predominantly as inhibitors of PKR activation under homeostatic conditions [40]. These inhibitory circRNAs are characterized by short, imperfect intramolecular RNA duplexes, typically approximately 16–26 bp in length. These duplex regions are commonly flanked by single-stranded RNA segments, a structural arrangement that permits PKR binding but fails to support productive PKR dimerization and autophosphorylation [40]. circRNA binding to PKR effectively sets a threshold for activation. During viral infection or cellular stress, activation of the OAS–RNase L pathway leads to rapid degradation of circRNAs, releasing PKR from inhibition and enabling robust activation in response to infection or cellular stress [40].

Beyond PKR, evidence for circRNA engagement of other RNA sensors remains limited. Exosomal circ-*CBLB* has been proposed to engage and activate TLR3 when taken up by the endosome in models of Rheumatoid Arthritis [177]. However, whether this reflects direct circRNA-sensing or secondary effects of circRNA processing by endosomal ribonucleases into RNA fragments is unclear. In soft-tissue sarcomas, circ*Csnk1g3* and circ*Ankib1* were shown to bind RIG-I and inhibit type I IFN signalling, thereby limiting immune cell infiltration into the tumour microenvironment. This occurred in the context of elevated endogenous retroviral element transcription and cytosolic dsRNA accumulation, revealing circRNAs as modulators of tumour-associated innate immune activation [178]. Similarly, circ*HOMER1* was reported to bind both RIG-I and the RIG-I cofactor G3BP1, reducing RIG-I engagement with viral RNA and dampening antiviral responses *in vitro* and *in vivo*. Intriguingly, RIG-I itself can give rise to a circRNA (circ*RIG-I*), which interacts with DDX3X to promote inflammation and tumour progression in colorectal cancer, indicating possible feedback loops between RNA sensors and circRNA biogenesis [179].

Engineered circRNAs generated from T4 bacteriophage or by Anabaena pre-tRNA group I intron can activate PKR and/or RIG-I [180]. By contrast, circRNAs generated through canonical mammalian spliceosome-dependent back-splicing that relies on complementary *Alu* elements or use of a T4 RNA ligase were immunologically inert [180]. Importantly, masking the phage intron with mammalian intronic sequences abolished RIG-I activation, suggesting that the splicing mechanism itself, rather than circularity *per se*, imprints immunogenic features on circRNAs [180]. Understanding the features that enable circRNAs to inhibit PKR, and potentially other RNA sensors, is of considerable interest. Exogenous circRNA treatment has recently been shown to be protective in models of several inflammatory diseases, including systemic lupus erythematosus, Alzheimer's disease [181] and osteoarthritis [182], raising the possibility that circRNAs may represent a novel therapeutic modality.

## 9. Avoiding self-detection: regulating nuclear RNA retention, export and decay

For a cellular dsRNA to be perceived as a threat by the cytoplasmic sensing system, it must first be present in the cytoplasm, yet the nucleus is the principal site at which potentially immunostimulatory dsRNA is generated. Therefore, selective nuclear retention, regulated export and nuclear RNA decay constitute a primary regulatory layer of self-tolerance that constrains the duplex RNA reaching the sensors. However, the evidence that these processes directly govern sensing is largely correlative rather than direct.

The clearest molecular link between nuclear sequestration and suppressed dsRNA immunogenicity is that hyper-edited, inosine-rich dsRNAs are retained in the nucleus by a complex containing the inosine-specific binding protein p54nrb (NONO), the splicing factor PSF (SFPQ) and the inner-nuclear-matrix protein matrin 3 [183]. Because the inosine marks that destabilize duplex structure and impair MDA5 filament formation also license retention, A-to-I editing acts simultaneously as a molecular tag of self and potentially a determinant of subcellular localization [183]. The physical site of retention is the paraspeckle, a nuclear body scaffolded by the long non-coding RNA *NEAT1* and assembled on the DBHS proteins NONO, SFPQ and PSPC1, which sequesters hyper-edited and inverted-repeat-containing transcripts. Consistent with a gatekeeping function, depletion of *NEAT1* and the resulting loss of paraspeckle integrity re-localizes nuclear-retained mRNAs bearing inverted repeats to the cytoplasm. A central caveat, however, is that these studies established control of cytoplasmic export and of aberrant protein production but did not test whether the re-localized RNA was sensed. Attempts to test the hypothesis by perturbing paraspeckles are confounded by the independent functions of their protein components: depletion of SFPQ induces type I IFN and MDA5 expression in uninfected cells, but SFPQ associates with ADAR1 and modulates A-to-I editing, so its depletion alters editing as well as RNA localization, and the IFN response cannot be attributed cleanly to release of retained dsRNA from the nucleus [184]. Also, the paraspeckle-retained transcripts are themselves hyper-edited and therefore poor MDA5 ligands, so their release would not necessarily be immunogenic; retention and editing may be partly redundant safeguards acting on the same substrate rather than independent checkpoints.

In our own recent work, a genome-wide screen identified GGNBP2, acting between dsRNA transcription and its cytoplasmic sensing by MDA5. Loss of GGNBP2 prevented induction of type I IFN following loss of ADAR1 editing by altering the subcellular distribution of endogenous A-to-I editing substrates and reducing the cytoplasmic dsRNA load [152]. Importantly, GGNBP2 provides genetic and functional evidence that redistributing editing substrates away from the cytoplasm can limit MDA5 activation, but it does not resolve the underlying mechanism. Whether GGNBP2 acts on the transcription, processing, retention, export or decay of these substrates remains undefined [152].

Whether a given nuclear transcript is exported or instead retained and degraded is actively arbitrated by the export machinery. Bulk messenger RNA export proceeds through the NXF1 (TAP)–NXT1 receptor together with the TREX complex and its adaptor ALYREF, and the nuclear exosome cofactor MTR4 (MTREX) competes directly with ALYREF for binding at the 5′ cap, partitioning individual transcripts between productive export and exosomal degradation. At a systems level, the kinetics of nuclear export themselves shape the innate response, with the rate of mRNA export tuning the expression of innate immune response genes. Importantly, export kinetics in this context govern the transcript levels of downstream interferon-stimulated output genes rather than the upstream endogenous dsRNA ligands themselves. Thus, nuclear export rate-limits the magnitude of the inflammatory response rather than the size of the primary immunostimulatory dsRNA pool.

Nuclear RNA decay provides the complementary safeguard, eliminating aberrant and structured transcripts before they can be exported. The principal nuclear 3′→5′ exonuclease is the RNA exosome, directed to its substrates by two adaptor complexes built around the helicase MTR4 (MTREX): the nuclear exosome targeting (NEXT) complex, comprising MTR4, ZCCHC8 and RBM7, which acts on short non-polyadenylated species such as promoter-upstream and enhancer RNAs; and the poly(A)-tail exosome targeting (PAXT) complex, comprising MTR4, ZFC3H1 and PABPN1, which targets longer polyadenylated and prematurely terminated transcripts [185]. This nuclear helicase MTR4, encoded by *MTREX* (formerly SKIV2L2), is a distinct paralogue of the cytoplasmic SKI-complex helicase SKIV2L, the two enzymes feeding the nuclear and cytoplasmic exosomes, respectively. When nuclear surveillance is impaired, for example by depletion of MTR4 or ZFC3H1, normally unstable nuclear transcripts, including prematurely terminated and cryptically processed RNAs, escape decay and accumulate in the cytoplasm and repress translation [186]. However, this was thought to be owing to sequestration of ribosomes, rather than activation of dsRNA sensors like PKR or OAS/RNaseL that inhibit translation, so further work is needed to clarify this mechanism.

Viewed together, the principle that the nuclear handling of dsRNA and its precursors can regulate cytoplasmic sensing is plausible, although direct evidence of disrupted nuclear processing activating sensors is largely absent. Several questions therefore remain open: It is unresolved whether retention is causally upstream of sensing or merely correlated with it, because no genetic tool has yet separated the localization of a dsRNA from the transcriptional and editing activities of the proteins that retain it. Finally, the mechanism by which factors such as GGNBP2 redistribute editing substrates, and whether they act through export, retention or decay, is undefined and represents a tractable next step [152]. Resolving these points will require experiments that manipulate the subcellular location of a defined dsRNA while holding its editing status and abundance constant, and that read out each sensor arm independently.

## 10. Avoiding self-detection: regulating sensor proteins’ ability to identify or engage dsRNA

Cellular dsRNAs also evade recognition when cellular RNA-binding proteins bind, modify or sequester duplexes away from innate immune sensors. Beyond its catalytic role in editing, cytoplasmic ADAR1p150 directly competes with sensors by binding and sequestering cellular dsRNAs. This non-catalytic shielding is sufficient to suppress PKR activation, a property unique to ADAR1p150 that cannot be rescued by the nuclear-restricted ADAR1p110 isoform [89,187]. This indicates that competition for dsRNA between both sensor proteins and other RNA-binding proteins can modify the tolerance to cellular dsRNAs. In contrast to the ADAR1p150-PKR competition model, an additional mechanism was recently described for how dsRNA-sensing can be moderated [95,188]. In this model, the protein PACT acts to prevent PKR from scanning along the dsRNA that PKR can access in the cytoplasm, rather than binding and sequestering the dsRNA, thus inhibiting PKR molecules from interacting with another PKR molecule to allow phosphorylation and activation.

This multi-level shielding extends beyond ADAR1p150 and PACT to include abundant RNA helicases that limit the pool of endogenous dsRNA before it can engage a sensor. The nuclear DExH-box helicase DHX9 binds specifically to inverted-repeat *Alu* elements (*IRAlus*) transcribed within genes and unwinds them, and its loss leads to the accumulation of dsRNA, aberrant circular RNA production and translational repression of *IRAlus*-containing transcripts [189]. Furthermore, combined loss of DHX9 and ADAR1 induces a viral-mimicry response in which endogenous dsRNA activates cytosolic sensing [190]. The related DEAD-box helicase DDX3X performs an analogous function, preventing the aberrant cytoplasmic accumulation of endogenous dsRNA and interacting with ADAR1p150 to promote A-to-I editing, so that its loss triggers MDA5-dependent type I IFN and synergizes with loss of ADAR1 [191]. DDX3X is, however, dual-function, as it is also a positive component of antiviral signalling downstream of the sensors within the TBK1-dependent pathway [192]; its net effect is therefore context-dependent, and it illustrates that a single helicase can both restrict the endogenous trigger and amplify the response once sensing has occurred.

Other dsRNA-binding proteins shield duplexes by occupying or masking them rather than by resolving them. Staufen1 (STAU1) binds 3′UTR *IRAlus* and, much like ADAR1p150, precludes PKR binding to the same duplexes, thereby preventing PKR activation and the resulting eIF2α phosphorylation and translational shutdown [193]. As with several of these factors, the role of STAU1 is context-dependent, as in other settings it stabilizes endogenous retroviral and SINE dsRNAs and thereby enhances, rather than suppresses, the IFN response [194]. Shielding can also be highly sequence-specific: the signal recognition particle proteins SRP9 and SRP14 bind the *Alu* domain of the abundant, 5′-triphosphorylated RNA polymerase III transcript 7SL and mask it from RIG-I, and when the SRP9/14-to-7SL ratio falls, unshielded 7SL is loaded into exosomes and activates RIG-I in recipient cells, coupling stromal activation to pattern-recognition-receptor signalling in cancer [80]. Likewise, elevated transcription of 7SL by RNA polymerase III can also lead to accumulation of unshielded 7SL, activating RIG-I and enhancing anti-viral activity [195].

These examples define a repertoire of distinct mechanisms through which occupancy of endogenous dsRNA by RNA-binding proteins helps to set the threshold for its recognition: duplex resolution by DHX9 and DDX3X, steric sequestration or occlusion by ADAR1p150 and STAU1, structural masking by SRP9/14, and interference with sensor scanning by PACT. That several of these proteins, including DDX3X and STAU1, can either restrain or promote sensing depending on context underscores that this threshold reflects a dynamic balance rather than a fixed hierarchy.

## 11. Avoiding self-detection: cytoplasmic RNA degradation

Analogous to the nuclear control pathways, cytoplasmic RNA decay machineries act as another layer of defence against expressed retroelements. Cells employ multiple RNA degradation pathways to prevent the accumulation of endogenous dsRNAs that would otherwise resemble viral molecules and activate innate immune sensors.

The 5′→3′ exonuclease XRN1 plays a critical role in degrading retroelement-derived and viral RNAs. It was identified as the top co-dependent gene with ADAR1 across cancer cell lines, suggesting they may act in a common pathway [47]. Supporting a role in dsRNA control, XRN1 exonuclease activity limits dsRNA accumulation during Vaccinia virus (VacV) infection, preventing activation of host dsRNA sensors [196]. In the absence of XRN1, dsRNA accumulates, activating the dsRNA sensors and reducing viral replication. Cancer cells with elevated type I IFN signalling are acutely sensitive to depletion of XRN1 owing to accumulation of endogenous dsRNA and activation of MAVS-dependent IFN signalling and PKR [47]. Cell lines that were resistant to XRN1 depletion became sensitive following treatment with demethylating agents that elevate dsRNA expression, supporting the concept that multiple layers of regulation synergize to dampen immune responses to cellular RNA.

The RNA helicase SKIV2L (also known as SKI2) is a cofactor of the cytoplasmic RNA exosome, which is responsible for 3′→5′ degradation of RNAs. SKIV2L unwinds secondary structures to feed RNAs into the exosome, facilitating degradation of cytosolic RNAs. Defects in SKIV2L cause hyperactivation of RIG-I, although the mechanism was unclear until recently. SKIV2L loss amplified IFN responses to exogenous ligands such as poly(I:C) or viral RNA [62]. Mechanistically, the dsRNA ligand activated OAS-RNaseL, leading to degradation of cellular RNAs by the exosome, which then amplified MAVS-dependent IFN production via RIG-I. Interestingly, cells expressing a p.A76V OAS1 gain-of-function variant, which causes an autoinflammatory immunodeficiency via activation of OAS, independent of a dsRNA ligand, were also more sensitive to loss of SKIV2L [62,65,77]. Knock-out of RNaseL, MAVS or RIG-I prevented the hyperactivation of IFN in OAS1^A76V^SKIV2L^KO^ cells, whereas deletion of the IFN receptor, IFNAR1, did not modify the phenotype. The data suggest that RNA degradation via SKIV2L prevents accumulation of RIG-I-activating dsRNA ligands, downstream of RNaseL activation. However, direct evidence for the involvement of the RNA degradation machinery in this pathway was lacking, and it remains possible that this is a function of SKIV2L, independent of RNA degradation. Biallelic mutations in SKIV2L cause a severe autosomal recessive multisystem disorder called THES2, including excess type I IFN signalling and immunodeficiency, supporting a role in innate immune sensing pathways [78].

Maharana *et al.* [197] demonstrated that SAMHD1, a dNTPase mutated in Aicardi-Goutières syndrome, normally prevents accumulation of immunogenic ssRNA. In SAMHD1-deficient cells, excess ssRNA dissolves RNA–protein condensates that otherwise sequester dsRNA. The release of dsRNA from these condensates activates MDA5 and downstream IFN signalling. This study shows that RNA degradation safeguards extend beyond direct dsRNA clearance and that by controlling ssRNA levels, SAMHD1 indirectly regulates the exposure of dsRNA to innate sensors. Thus, RNA decay pathways and RNA-protein condensate dynamics converge to prevent sterile activation of antiviral immunity.

Together, these studies establish that RNA degradation machineries act at multiple levels to prevent immunogenic dsRNA accumulation. When these pathways are disrupted, dsRNA accumulates and triggers IFN responses, underscoring their essential role in maintaining immune homeostasis.

## 12. Mistaken identity: rare human genetic diseases highlight cellular-derived RNAs as immunogenic stimuli

In the context of viral infections, as noted above, there is evidence of direct interaction between the foreign dsRNA, as well as host dsRNA, and the cytoplasmic sensors. There are multiple diseases associated with sterile activation of cytoplasmic dsRNA sensors, highlighting that cellular RNAs may be sensed and can be initiators of pathogenesis in these contexts. Analysis of rare monogenic diseases has identified patients with variants in the cytoplasmic RNA sensors themselves that have been highly informative for understanding the sensing of cellular dsRNA species. For example, germline variants in *IFIH1* (MDA5), *DDX58* (RIG-I), *TLR7* and *UNC93B1* have all been reported in patients with autoimmune/autoinflammatory diseases including Aicardi-Goutières syndrome, Singleton-Merten syndrome, SLE and paediatric neuroinflammatory syndromes [20–22,70,72,73,198]. The delineation of the genetics of these human diseases has provided context for understanding how cellular RNAs may be inappropriately sensed and trigger disease.

Murine models have complemented the human genetics to enhance our understanding of the *in vivo* functions of both the RNA sensors and in determining the pathways specifically impacted by the different RNA sensors. Loss-of-function studies, predominantly protein-deficient models, were developed for many of the cytoplasmic RNA sensors. The loss of *Ifih1* (Mda5) [10,24], *Eif2ak2* (Pkr) [41] and *Rnasel* (RNaseL) [42] all resulted in viable gene-deficient mice. These mice were largely normal when housed conventionally and not challenged. Loss of *Ddx58* (RIG-I) revealed a more complex phenotype, with one deficient allele displaying a high level of embryonic lethality and a second recovering viable deficient mouse [17,18]. While these alleles have enabled a clear understanding of the requirement for each of these sensors in sterile and infection settings, these murine models often do not recapitulate the human diseases where variants in these genes are observed, and a protein product is still expressed.

More recent models have sought to recapitulate the human variants in mice or to develop point mutant models that more closely approximate patient genetics where variant proteins are expressed, rather than complete protein deficiency, as was applied in the first-generation murine models. Models of *IFIH1* gain-of-function polymorphisms indicated that these resulted in a heightened type I IFN response, indicative of an elevated response to cellular dsRNAs species [26]. Variants are frequently associated with the ATPase domain and RNA-binding interface of the protein [8]. These increase the affinity of the protein for imperfectly paired dsRNA structures by decreasing the efficiency of ATP-driven filament disassembly [23,199]. Similarly, gain-of-function MDA5 mutations in mouse models resulted in inflammatory phenotypes consistent with the effects in patients [27].

Recent work has suggested MDA5 genetic variants offer a direct fitness trade-off between viral clearance and autoimmune tissue damage [32]. Loss-of-function variants protect against autoimmune diseases including type I diabetes, psoriasis and hypothyroidism but at the cost of increased risk for inflammatory bowel disease associated with reduced viral defence (reviewed in [32]). A recent mouse model of *DDX58-variant-associated* disease has enabled the identification of cellular ligands that are sensed by the mutant RIG-I protein [16]. Within the kidney, relevant to the nephritis phenotype both in mice and patients, mutant RIG-I is activated by Y-RNA, most prominently RNY4 [16]. Y-RNAs are a class of small, non-coding RNAs involved in a variety of processes including DNA replication, RNA processing and cellular stress responses. Intriguingly, while mutant RIG-I was activated by RNY4, wild-type RIG-I was not, suggesting that the mutation in RIG-I shifts the substrate preference for cellular RNAs and that this is important in initiating the pathogenic response [16]. Similar observations have been made with WT and mutant MDA5, and activation by cellular paired *Alu* repeats [23]. Collectively, the combination of human genetics and confirmatory murine modelling has demonstrated that inappropriate and aberrant sensing of cellular RNA species is a key driver of types of autoinflammatory diseases. While variants in RNA sensor proteins themselves are a genetic cause of autoinflammatory syndromes, these illustrate that homeostasis must actively maintain safeguards against the aberrant sensing of cellular dsRNAs in the cytosol.

## 13. Beyond rare disease and how this information can be applied

Maintaining immune homeostasis requires tight control over the biogenesis and subcellular localization of endogenous dsRNA [2]. However, when pathogenic variants in regulatory or sensor proteins disrupt this equilibrium, cells mistake ‘self’ for ‘non-self’. Variants that disrupt transcription, RNA splicing, RNA export and RNA modifications that increase or mislocalize cellular dsRNA can lead to inflammation and disease [2]. This has historically been associated with rare inherited disease, with the type I interferonopathies providing an example [22]. In the case of type I interferonopathies like Aicardi-Goutières syndrome, variants result in profound and debilitating mistakes in ‘self’–‘non-self’ discrimination with systemically elevated type I IFN and severe impacts on the patient and shortening of lifespan [21,200]. These rare diseases powerfully demonstrate the importance of the cellular pathways being able to efficiently and specifically differentiate between cellular RNAs and foreign and pathogenic RNA. However, it is now beginning to be appreciated that these same pathways may contribute to common forms of inflammatory and autoimmune diseases. Consistently, dysregulated cellular dsRNA homeostasis has been implicated in more common inflammatory diseases, including psoriasis and SLE [201], Alzheimer's disease [202] and amyotrophic lateral sclerosis (ALS) [203,204]. This emerging evidence suggests that dysregulated dsRNA is a shared feature across a diverse disease spectrum with an inflammatory component, often involving neurological dysfunction [3]. In many of these diseases, it appears that excessive activation of these pathways in a sterile setting contributes to the disease. This emphasizes the need to understand how these pathways are activated—both the nature and specifics of the ligands and the protein-protein and protein regulatory pathways that govern activation and deactivation of the sensors/signalling—as areas to consider when trying to devise new therapies for these patients. Defining the mechanisms that regulate cellular dsRNA homeostasis is therefore of broad and urgent importance with direct implications for patients where variants in these pathways result in profound illness and shortened lifespan, but also as potential significant contributors to chronic autoinflammation and autoimmune diseases [2].

Delineation of these pathways has been key to understanding how cellular RNAs can be shielded from or exposed to sensing by the cytoplasmic RNA-sensing machinery. There is evolving evidence that cellular dsRNAs are regulated at multiple levels to moderate their immunogenicity and achieve ‘self’-tolerance. The system does not have to eliminate all immunogenic cellular species, just retain enough active control mechanisms to keep the levels below a yet undefined threshold level that leads to triggering of the innate immune response. Loss of one or more of these tolerance mechanisms, such as variants in *ADAR*, lowers the threshold set point and can lead to aberrant self-sensing. Such a model is consistent with a continuous level of basal sampling and sensing of the cellular RNA environment, and that the innate immune response is only activated once the levels of RNAs in this pool diverge sufficiently from homeostatic populations that the buffering mechanisms cannot restrict access of the RNA sensor proteins to the cellular RNAs in the cytoplasm.

This also highlights potential applications of this knowledge where driving activation of these pathways may be beneficial. Most prominently among these is the desire to manipulate and enhance the response to cellular RNA species for therapeutic gain, particularly in cancer [140,205,206]. Pharmacological induction of viral mimicry has emerged as a key approach proposed to be able to convert immunologically ‘cold’ or treatment-resistant tumours into immune-reactive (‘hot’) tumours [132,207]. Many malignancies maintain immune evasion by hypermethylating ERV loci and suppressing baseline IFN signalling. Low-dose epigenetic therapy, such as DNMT and HDAC inhibitors, can mediate selective de-repression of these silenced ERVs. The resulting activation of the cytoplasmic RNA-sensing response, primarily attributed to the MDA5-MAVS axis, triggers cell-intrinsic type I IFN production, upregulates MHC class I antigen presentation and induces chemokines (such as CXCL9 and CXCL10) that recruit cytotoxic T lymphocytes into the tumour microenvironment. By artificially reprogramming the local inflammatory landscape, viral mimicry reverses primary resistance and strongly synergizes with immune checkpoint blockade therapies, including anti-PD-1 and anti-PD-L1 agents. Consistent with this, a recent study showed that clinically approved SARS-CoV-2 mRNA vaccines can similarly induce a transient type I IFN response, activate innate immunity, enhance antigen presentation and CD8⁺ T-cell priming and sensitize otherwise immunologically cold tumours to immune checkpoint blockade in mouse models [208]. These findings were supported by multiple large retrospective clinical cohorts, in which receipt of an mRNA vaccine within 100 days of initiating immune checkpoint blockade was associated with significantly improved overall survival in patients with melanoma and non-small cell lung cancer, including those with immunologically cold tumours [208]. These studies indicate that the nonspecific systemic stimulation of type I IFNs by vaccines containing mRNA can also prime tumour environments for immune-mediated killing.

Directly leveraging intracellular dsRNA-sensing pathways as a treatment modality in cancers has become increasingly supported by genetic data derived from screens in cancer cells [3,162]. Key targets include the A-to-I RNA editing enzyme ADAR1, supported by extensive genetics, which indicates that inhibition of editing or RNA binding by ADAR1p150 will drive activation of MDA5 and PKR [89,209–212]. This has been demonstrated to substantively enhance the response of tumours deficient in ADAR1 to immune checkpoint blockade (‘immunotherapy’) [209–211]. The detailed understanding of these pathways now enables prediction of the RNA-sensing pathways that will be activated upon loss of specific functions of ADAR1 [89,90]. Activating ZBP1 has also emerged as a potential means to activate the cellular response to self-nucleic acids, although in the case of ZBP1 both RNA and DNA could be mediating the sensitization reported [163]. Another example of an RNA-modifying enzyme as a therapeutic target that has progressed further than ADAR1 is the inhibition of the m^6^A writer METTL3, where small molecule development has progressed, and these compounds appear to be exerting their effect by promoting tumour and tumour-infiltrating cells to sense cellular-derived RNA species [166,213,214]. We will have to await the results from the clinical trials and the development of new generations of compounds that can target a range of RNA-modifying pathways to more completely appreciate the potential that manipulation of self-sensing holds as a treatment in cancers.

## 14. Summary

The ability for a cell to be able to determine the origin of dsRNA in the cytoplasm is essential for normal homeostasis and protection against pathogens. Cells have evolved a range of mechanisms that enable cellular-derived dsRNA to be managed, from restricting expression and silencing to actively modifying the sequence and structure of expressed RNA through complementary and cooperative mechanisms including direct RNA modification, splicing and degradation (table 2). These processes have evolved to promote self-tolerance, allowing the immune system to avoid inappropriate responses to repetitive genetic elements that have accumulated over evolutionary time. These mechanisms normally serve to allow efficient differentiation between cellular dsRNA and foreign dsRNA, enabling the cytoplasmic innate immune system to provide effective protection. However, we are increasingly appreciating that changes, through mutations or disruptions to homeostasis, across these pathways can lead to a range of both rare and common diseases associated with inflammation and type I IFN. Growing evidence indicates that cellular transcripts act as ligands for cytoplasmic sensors. Disrupted cellular homeostasis, independent of viral infection, can alter the transcriptome to generate immunogenic ligands. This would greatly broaden the potential functions of these sensors across diverse disease settings and suggest that the targeted manipulation of either the cellular transcriptome or sensing responses could be prospectively used therapeutically. These pathways are already being explored in cancer or as a means of priming for vaccination. The continued understanding of these pathways and how they interact and cooperate will provide both a deeper understanding of human disease and highlight areas of opportunity that can be leveraged to develop better ways to treat disease.

**Table 2.** Key checkpoints in endogenous dsRNA self-tolerance.

**checkpoint layer**	**key molecular components**	**primary target transcripts**	**sensor pathway suppressed**
epigenetic silencing	SETDB1 [45], TRIM28 [128], HUSH [129] and DNMTs [132]	ERVs, LINEs and LTRs [130]	MDA5 and PKR
RNA modification	ADAR1 (p150/p110) [89,150], METTL3 [165,167] and METTL14	inverted *Alu*s, B1/B2 SINEs and mRNAs [148]	MDA5, PKR, ZBP1 and OAS [90,162]
splicing surveillance	SF3B1 [170], hnRNPC [171] and hnRNPM [172]	cryptic intronic repeats and LINEs [180]	MDA5, RIG-I and PKR [40]
nuclear retention and decay	NEXT, PAXT [186] and Paraspeckles (NONO and SFPQ) [183,184]	promoter-upstream RNAs and unedited IRs [185]	MDA5 and PKR
protein shielding	ADAR1p150 [89], PACT [95,188], STAU1 [193,194], DHX9 [189,190] and DDX3X [191,192]	inverted *Alu*s, 7SL RNA [195] and circRNAs	PKR, ZBP1 and RIG-I
cytoplasmic decay	XRN1 [47,196], SKIV2L [62,77] and SAMHD1 [197]	cleavage fragments and unshielded ssRNA/dsRNA	RIG-I, MDA5 and PKR

## Data Availability

This article has no additional data.
